# Dock10, a Cdc42 and Rac1 GEF, induces loss of elongation, filopodia, and ruffles in cervical cancer epithelial HeLa cells

**DOI:** 10.1242/bio.20149050

**Published:** 2015-04-10

**Authors:** Natalia Ruiz-Lafuente, María-José Alcaraz-García, Azahara-María García-Serna, Silvia Sebastián-Ruiz, María-Rosa Moya-Quiles, Ana-María García-Alonso, Antonio Parrado

**Affiliations:** Immunology Service, Virgen de la Arrixaca Clinic University Hospital, Institute for Biohealth Research (IMIB-Arrixaca), Ctra. Madrid-Cartagena s/n, El Palmar, 30120, Murcia, Spain

**Keywords:** Dock10, Dock9, Dock11, Cdc42, Rac1, filopodia, membrane ruffles

## Abstract

Dock10 is one of the three members of the Dock-D family of Dock proteins, a class of guanine nucleotide exchange factors (GEFs) for Rho GTPases. Its homologs Dock9 and Dock11 are Cdc42 GEFs. Dock10 is required for maintenance of rounded morphology and amoeboid-type movement. Full-length isoforms of Dock10 have been recently cloned. Here, we address GTPase specificity and GEF activity of Dock10. In order of decreasing intensity, Dock10 interacted with nucleotide-free Rac1, Cdc42, and Rac3, and more weakly with Rac2, RhoF, and RhoG. Inducible expression of Dock10 in HeLa epithelial cells promoted GEF activity on Cdc42 and Rac1, and a morphologic change in two-dimensional culture consisting in loss of cell elongation, increase of filopodia, and ruffles. Area in contact with the substrate of cells that spread with non-elongated morphology was larger in cells expressing Dock10. Inducible expression of constitutively active mutants of Cdc42 and Rac1 in HeLa cells also induced loss of elongation. However, Cdc42 induced filopodia and contraction, and Rac1 induced membrane ruffles and flattening. When co-expressed with Dock10, Cdc42 potentiated filopodia, and Rac1 potentiated ruffles. These results suggest that Dock10 functions as a dual GEF for Cdc42 and Rac1, affecting cell morphology, spreading and actin cytoskeleton protrusions of adherent HeLa cells.

## Introduction

Rho GTPases are small proteins involved in actin cytoskeleton organization, cell shape, adhesion and movement (Wennerberg and Der, 2004; Aspenström et al., 2004; Heasman and Ridley, 2008). The “classic” Rho GTPases cycle between two forms, GDP- or GTP-bound. The GTP-bound is the “active” form, because it associates with downstream effectors. There are 12 genes encoding “classic” Rho GTPases in mammals, which can be grouped structurally and functionally in Cdc42-related (*Cdc42*, *RhoJ/TCL*, and *RhoQ/TC10*), Rac1-related (*Rac1*, *Rac2*, *Rac3*, and *RhoG*), RhoA-related (*RhoA*, *RhoB*, and *RhoC*), *RhoD*, and *RhoF/Rif*. Additional 8 genes encode the “atypical” Rho GTPases, constitutively bound to GTP. The activity of Rho GTPases is regulated by 3 classes of proteins: 1) Guanosine nucleotide exchange factors (GEFs), which stimulate the weak intrinsic exchange activity of Rho GTPases to promote the formation of the GTP-bound form; 2) GTPase-activating proteins (GAPs), which stimulate GTPase activity and conversion to the GDP-bound form; and 3) Rho GDP dissociation inhibitors (GDIs), which retain the GDP-bound form in the cytoplasm.

Dedicator of cytokinesis (Dock) proteins are large proteins which constitute a major class, together with the Dbl-homology proteins, of Rho GEFs (Rossman et al., 2005; Meller et al., 2005). Dock proteins are characterized by the presence of a carboxy-terminal domain known as CZH2, where their GEF function resides. There are 11 *Dock* genes in mammals, grouped in 4 families: A, B, C, and D. The D, or Zizimin, family, characterized by an N-terminal pleckstrin homology domain, is composed of 3 members, *Dock9/Zizimin1*, *Dock10/Zizimin3*, and *Dock11/Zizimin2*. Dock9 and Dock11, and their CZH2 domains, interact and activate Cdc42 (Meller et al., 2002; Nishikimi et al., 2005; Lin et al., 2006). Weak interactions of the CZH2 domain of Dock10 with Cdc42 and RhoJ have been reported (Nishikimi et al., 2005), but specificity of the complete Dock10 protein is unknown.

Rho GTPases play different roles in actin cytoskeleton dynamics. Cdc42-related, and RhoD and RhoF proteins, induce filopodia; Rac1-related proteins induce lamellipodia and membrane ruffles; RhoA-related proteins induce stress fibers (Wennerberg and Der, 2004; Aspenström et al., 2004; Chhabra and Higgs, 2007; Heasman and Ridley, 2008). When cultured on planar substrata without a migration stimulus, fibroblasts adhere and spread to adopt an elongated shape, and move randomly. Protrusive activity is determined by local regulation of Rho GTPase activation. Crosstalk regulation between GTPases favors their coordination. Thus, Cdc42 contributes to Rac1 activity (Nobes and Hall, 1995; Yang et al., 2006), Cdc42 and RhoG contribute to lamellipodia formation through Rac proteins (Monypenny et al., 2009), and Rac1 downregulates filopodia formation (Steffen et al., 2013).

We previously reported cloning of the full length coding sequences of the human and mouse *Dock10* genes (Yelo et al., 2008; Alcaraz-García et al., 2011). Two isoforms, designated Dock10.1 and Dock10.2, arise from alternative transcription start site usage. Expression of Dock10 is prominent in lymphoid organs, being T lymphocytes enriched in Dock10.1 and B lymphocytes in Dock10.2. Interleukin 4 upregulates Dock10 expression in B lymphocytes. Dock10 expression is also upregulated in aggressive cases of papillary thyroid carcinomas (Fluge et al., 2006), and in the epithelial to mesenchymal transition of squamous carcinoma cells (Humtsoe et al., 2012). What we know about the role of Dock10 comes from a single study using gene silencing, showing Dock10 as a factor that sustains the rounded morphology and amoeboid-type movement in melanoma cells (Gadea et al., 2008).

In this paper, we aimed to investigate Dock10 function by defining, for the first time, the specificity of the complete Dock10 protein for “classic” Rho GTPases, and studying its effects in human HeLa cells, using stable inducible expression. Our results show that Dock10 interacts with and activates Cdc42 and Rac1. Dock10 promotes a morphological transition from polygonal elongated to more rounded, non-polygonal cells. These cells develop abundant filopodia, frequently spread their area in contact with the substrate while retaining the non-elongated shape, and had increased ruffling activity. These results suggest that Dock10 is a GEF with broader specificity than its zizimin homologs, targeting Cdc42 but also Rac proteins.

## Materials and Methods

### Cell lines

Human embryonal kidney (HEK) 293T cells, monkey kidney COS-1 cells, and human cervix carcinoma epithelial HeLa cells, were cultured on plastic flasks in Dulbecco's minimum essential medium supplemented with 10% Fetal Calf Serum (FCS; Biowhittaker, Cambrex, East Rutherford, NJ), 50 U/ml penicillin, 50 U/ml streptomycin, 2.5 µg/ml amphotericin B, and 2 mM l-glutamine (“complete medium”, CM) at 37°C in a humid atmosphere of 5% CO_2_. The three cell lines grow as monolayers with fibroblast-like morphology, and were maintained subconfluent by detachment with trypsin 0.05%-EDTA 0.02% in PBS (EuroClone, Milan, Italy) and routine subculture.

### *In vitro* interaction assays

GTPases binding assays were performed by GST pull-down experiments. *E. coli* BL21 DE3 cells transformed with plasmid constructs for inducible expression of N-terminally GST bound Cdc42, Rac1, Rac2 (generated from plasmid published in Hoppe and Swanson, 2004), Rac3 (generated from plasmid published in Hajdo-Milasinović et al., 2007), RhoA, RhoD (generated from plasmid published in Roberts et al., 2008), RhoF-SAAX (generated from a plasmid given by H. Mellor, University of Bristol, UK), RhoG-SAAX, RhoJ, and RhoQ (Neudauer et al., 1998) proteins, or GST alone, were grown in LB medium with 125 µg/ml of ampicillin and treated with 0.5 mM IPTG for 3 h. The plasmids used in this study, and the procedures to generate them, are listed in supplementary material Table S1. HEK 293T cells were transfected for 24 h with plasmid constructs for transient expression of FLAG-Dock9 (Meller et al., 2004), Dock10.1, HA-Dock10.1, Dock10.2, HA-Dock10.2 (generated from plasmids published in Alcaraz-García et al., 2011), Dock11, and HA-Dock11 (generated from plasmid published in Lin et al., 2006), using lipofectamine reagent (Invitrogen), following the manufacturer's instructions. Bacterial pellets were resuspended in Lysis Buffer A containing 50 mM Tris⋅Cl pH 7.5, 150 mM NaCl, 5 mM MgCl_2_, 1 mM DTT, 1% Triton X-100, proteinase inhibitor cocktail cOmplete, EDTA free (Roche, Basel, Switzerland), and 100 mg/ml lysozyme, and sonicated. Bacterial lysates were cleared by centrifugation and bound for 1 h on glutathione-sepharose 4B beads (GE Healthcare, Little Chalfont, UK). Beads were then washed in Tris Wash Buffer A containing 50 mM Tris⋅Cl pH 7.5, 150 mM NaCl, 5 mM MgCl_2_, 1 mM DTT, 0.5% Triton X-100, and cOmplete EDTA free, and preserved at −80°C in Tris Wash Buffer A with 10% glycerol. Protein loading in beads was quantified in Coomassie stained SDS-PAGE gels using BSA standards. A volume of beads containing 200 µg of loaded recombinant protein was washed three times and resuspended in 1 ml of either Solution A [20 mM Tris⋅Cl pH 7.5, 50 mM NaCl, 10 mM EDTA, 1 mM DTT, 0.1% Triton X-100, cOmplete EDTA free, and 5% glycerol (nucleotide-depleted)], Solution B [same as Solution A but with 1 mM GDP (Sigma-Aldrich, St-Louis, MI) and 10 mM MgCl_2_ instead of EDTA (GDP-loaded)], or Solution C [same as Solution B but with 1 mM GTP (Sigma-Aldrich) instead of GDP (GTP-loaded)]. Total protein extracts of transfected 293T cells (200 µl) were added to beads and incubated with end-over-end shaking at 4°C overnight. Beads were then washed again, and SDS loading buffer was added. Proteins were denatured for 10 min at 100°C, loaded onto SDS-PAGE gels and immunoblotted using HA, FLAG, or Dock specific antibodies. Antibodies used in this work and their dilutions for different uses are listed in supplementary material Table S2.

### Generation of stable cell clones with regulatable Dock10 expression

Stable clones with regulatable HA-Dock10 expression of HeLa cells were generated using the tet-off system following a procedure previously described with modifications (Parrado et al., 2000). HeLa-tTA cell clones were generated by transfection with the pUHD-15-1-Puro plasmid (Bernardo et al., 2007), using lipofectamine, and selected in 150 mm Falcon Tissue Culture-treated dishes (Corning Inc., Corning, NY) using CM supplemented with 1 µg/ml puromycin during 2–3 weeks. Colonies were gently scratched and aspired using micropipette points, and reseeded in 96-well plates filled with CM supplemented with puromycin. Isolated colonies were grown and regulation of transactivation by doxycycline (dox) was checked using the reporter plasmid pUHC-13-3, which drives expression of luciferase under the control of a promoter inducible by tTA (Gossen and Bujard, 1992). Dox binds and inactivates the tTA. The clone that best regulated transactivation by dox, designated HeLa-tTA, was subsequently transfected with the pJAG4-HA-Dock10 plasmid, which drives expression of HA-tagged Dock10 under the tTA-inducible promoter (generated by PCR cloning of Dock10 into pJAG4, a modified version of pJEF4; Parrado et al., 2000). HeLa cell clones were isolated during 2–3 weeks in CM with 1 µg/ml puromycin, 0.5 mg/ml G418, and 2 ng/ml dox, and colonies were grown following the same procedure as above with the same media used for selection. Colonies were checked for HA-Dock10 expression by western blot analysis, using extracts from replicate aliquots of cells washed free of dox and reseeded in CM either containing 2 ng/ml dox or lacking dox. One of the positive clones, C33, followed a third round of transfection either with pJAG2-EGFP-Cdc42Q61L or with pJAG2-EGFP-Rac1Q61L (generated from plasmids published in Subauste et al., 2000), which drive expression of the EGFP-tagged Cdc42 or Rac1 constitutively active mutants under the tTA inducible promoter. HeLa cell clones were isolated during 2–3 weeks in CM with 1 µg/ml puromycin, 0.5 mg/ml G418, 10 µg/ml zeocin and 2 ng/ml dox. The HeLa-tTA subline was subjected to a second round of transfection with constitutively active mutant GTPase plasmids and selection with puromycin, zeocin and dox. Single clones expressing EGFP-bound Cdc42 or Rac1 and double clones expressing HA-Dock10.1 and the EGFP-bound Cdc42 or Rac1 were identified after washing the cells free of dox in the selection dish, and culturing for 24 h in the absence of dox. Positive colonies were detected by green fluorescence using a JuLI™ Smart Fluorescence Cell Analyzer (NanoEnTek Inc, Seoul, Korea), then collected and grown following the same procedure as above with the same media used for selection. Colonies were grown and checked for expression of the GTPases western blot analysis as explained above.

### Cdc42/Rac activation assays

When bound to GTP, Rho GTPases interact with their effectors (Aspenström, 1999). PAK1 interacts with Cdc42·GTP and Rac1·GTP through its p21 binding domain (PBD). Replicate aliquots of the HeLa clones with regulatable expression of HA-Dock10 cultured for 24 h in the presence and absence of dox were assayed by pull down using GST-PAK1-PBD bound beads. Cell were lysed in Lysis Buffer B containing 50 mM Tris⋅Cl pH 7.2, 0.5 M NaCl, 10 mM MgCl_2_, 1% Triton X-100, and cOmplete EDTA free. GST-PAK1-PBD beads (40 µl) were added to cleared lysates (500 µl) and incubated with end-over-end shaking at 4°C for 1 h. Beads were then washed in Tris Wash Buffer B containing 50 mM Tris-Cl pH 7.2, 150 mM NaCl, 10 mM MgCl_2_, 0.5% Triton X-100, and cOmplete EDTA free, and SDS loading buffer was added. Proteins were denatured for 10 min at 100°C, loaded onto SDS-PAGE gels and immunoblotted using Cdc42 or Rac1 specific antibodies. In different control experiments, HeLa cells were treated with 100 ng/ml epidermal growth factor (EGF, Sigma-Aldrich), or HeLa protein extracts were incubated either with 1 mM GDP or 100 µM GTPγS (Sigma-Aldrich), a non hydrolyzable GTP analog.

### Western blot analysis

Proteins fractionated in SDS PAGE gels were electroblotted onto nitrocellulose membranes. Blots were blocked in TBST with 5% Difco™ skim milk (BD) for 1 h, then incubated with primary antibodies at the dilutions indicated in supplementary material Table S2 in TBST with 0.5% skim milk for 2 h. Following three washes of 5 min each in TBST, membranes were incubated with secondary antibodies in TBST with 2.5% skim milk for 1 h. After four final washes of 10 min each in TBST, immunoreactive proteins were detected using the Amersham ECL or ECL Plus Western Blotting Detection Reagents (GE Healthcare). Chemiluminescence images were acquired and quantitated in a Molecular Imager ChemiDoc™ XRS+ with Image Lab software (Bio-Rad Laboratories, Hercules, CA).

### Microscopy

HeLa cells were grown on to BioCoat collagen-coated chamber glass slides (Corning Inc.) and on to 12 mm BioCoat Poly-l-lysine-coated coverslips (Corning Inc.). Preparations were labelled using the F-actin visualization biochem kit (Cytoskeleton Inc., Denver, CO), following the manufacturer's instructions with minor modifications which consisted in the inclusion of incubation with anti-HA antibody for 1 h at 4°C after the permeabilization step, followed by three washes, and incubation with anti-rat-Alexa Fluor 488, 100 nM phalloidin-rhodamine (TRITC), and 1 µg/ml DAPI before final washing steps. Dako fluorescent mounting medium was placed between slides and coverslips. Cells were examined in an Eclipse T*i* inverted microscope (Nikon Instruments Inc., Melville, NY). Fluorescence images were acquired and analyzed using the NIS Elements software, including measures of cell area. Cell counting and classification in 4 subsets (polygonal elongated, non-polygonal/non-elongated, unclear/early spreading, and small round) were performed manually on F-actin labelled preparations. HeLa cells typically spread longitudinally and adopt a polygonal shape, with thick F-actin fibers delineating the cell edges. Some polygonal cells not having a strictly elongated appearance were also included in the group. The non-polygonal/non-elongated subset included cells that spread with a more rounded shape and lack stress fibers. Round cells included the mitotic, early post-mitotic or apoptotic cells, small and spherical. Last, the transitional group consisted of non-spherical cells with little spreading. Phase contrast and green fluorescent time lapse images of cells grown on to poly-l-lysine-coated coverslips were acquired in the Nikon Eclipse microscope. Coverslips were mounted in a heated stage circular chamber and cultured in buffer containing 10 mM HEPES, 147 mM NaCl, 2 mM KCl, 2 mM CaCl_2_, 1 mM MgCl_2_, 13 mM d-glutamine.

## Results

### Interaction of Dock10 with Rho GTPases

**Fig. 1.**
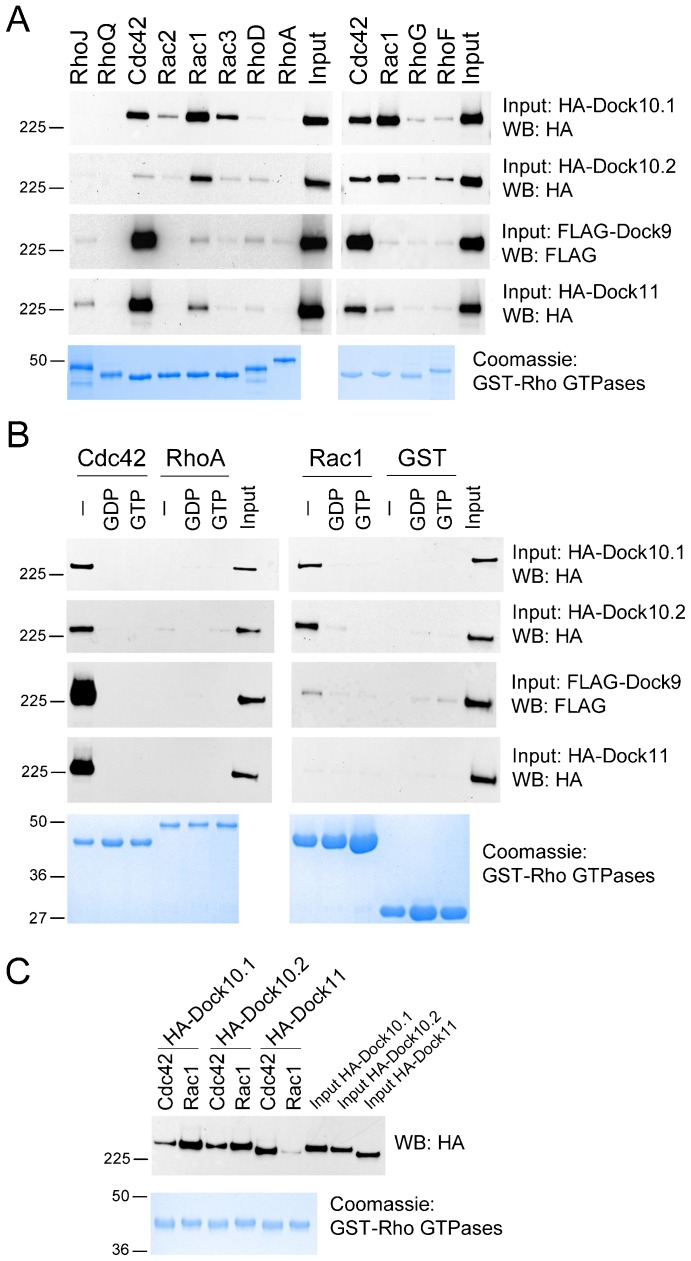
*In vitro* interactions of Zizimin proteins with Rho GTPases. (A) Interactions of Zizimin proteins, expressed in 293T cells, with the nucleotide-free forms of the “classic” Rho GTPases. GST pull-down assays using total protein extracts from 293T cells transfected with FLAG-Dock9, HA-Dock10.1, HA-Dock10.2, and HA-Dock11 and recombinant GST-bound Cdc42, Rac1, Rac2, Rac3, RhoA, RhoD, RhoF, RhoG, RhoJ, and RhoQ proteins. (B) As in A, but using the nucleotide-free forms, and the GDP- or GTP-loaded forms of GST-bound Cdc42, Rac1, and RhoA, and GST alone. (C) As in A, but loading only the HA-tagged proteins, HA-Dock10.1, HA-Dock10.2, and HA-Dock11, precipitated by the nucleotide-free forms of the GST-bound Cdc42 and Rac1. The position of the size markers are indicated in kDa to the left.

To determine the specificity of Dock10 for “classic” Rho GTPases, *in vitro* interaction assays were performed. Total protein extracts of 293T cells transfected with HA-Dock10.1, HA-Dock10.2, HA-Dock11, and FLAG-Dock9 were assayed for precipitation by GST-bound Cdc42, Rac1, Rac2, Rac3, RhoA, RhoD, RhoF, RhoG, RhoJ (TCL), and RhoQ (TC10). Using nucleotide-free forms of the GTPases, we found that both Dock10 isoforms interacted, in order of decreasing intensity, with Rac1, Cdc42, and Rac3, and weakly with Rac2, RhoF, and RhoG; Dock11 interacted with Cdc42, and weakly with Rac1, and RhoJ; and Dock9 interacted with Cdc42, and weakly with Rac1, and RhoD (Fig. 1A). The Zizimin proteins did not interact with GDP- or GTP-loaded Cdc42 or Rac1 (Fig. 1B). Similar results were obtained with non HA-tagged Dock10.1 and Dock10.2 (data not shown). Both Dock10 isoforms interacted with Rac1 with similar intensity, equivalent to that of Dock11 with Cdc42 (Fig. 1C). Interactions of both Dock10 isoforms with Cdc42, though slightly less intense than those with Rac1, were still much higher than those of Dock9 and Dock11 with Rac1. Therefore, results of our interaction assays are consistent with specificity of Dock9 and Dock11 for Cdc42, and dual specificity of Dock10 for Rac1 and Cdc42.

### Activation of Rho GTPases by Dock10.1

**Fig. 2.**
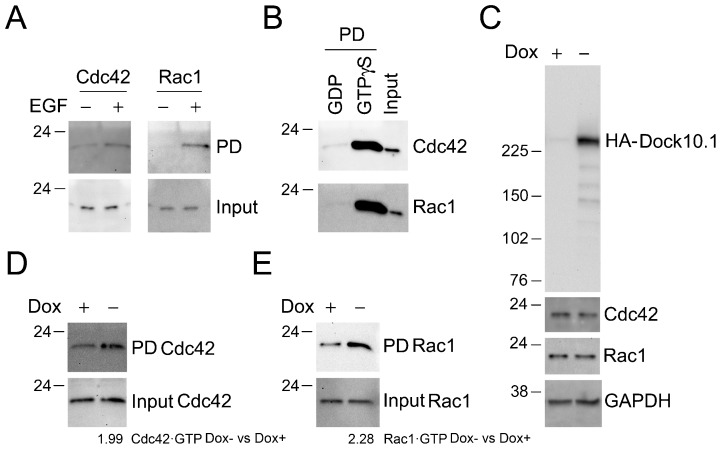
Activation of Cdc42 and Rac1 by Dock10.1. (A) Activation of Cdc42 and Rac1 induced by treatment with 100 ng of EGF per ml for 3 min in HeLa cells by GST-PAK1-PBD pulldown assays. (B) Activation of Cdc42 and Rac1 using GDP (negative control) and GTPγS treated HeLa extracts, by GST-PAK1-PBD pulldown assays. (C) Inducible expression of HA-Dock10.1 in a HeLa cell clone did not change the levels of expression of Cdc42 and Rac1. Total protein extracts were performed 24 h after washing the cells free of dox and reseeding with or without 2 ng/ml dox. (D,E) Activation of Cdc42 (D) and Rac1 (E) in the HeLa clone expressing HA-Dock10.1 by GST-PAK1-PBD pulldown assays using protein extracts obtained as in C. Numbers underneath the blots represent ratios between the Dox− and Dox+ signals, normalized by input, from pulled down GTPases·GTP. Inputs are depicted as loading controls for the assays in A,B,D,E. The position of the size markers are indicated in kDa to the left. PD, pulldown. Dox, doxycycline.

The ability of interacting *in vitro* with the nucleotide-free form, but not with the nucleotide-loaded forms of small GTPases, typifies the stages in GEF–small-GTPase interactions, where the GEFs stabilize the nucleotide-free form of the GTPase until GTP then displaces the GEF. To test the suggested role for Dock10 as a GEF for Cdc42 and Rac1, *in vitro* activation assays were performed by pulldown with GST-PAK1-PDB. Previously, we set up controls for this assay, such as a known model of Cdc42 and Rac1 activation by EGF in HeLa cells (Kurokawa et al., 2004 (Fig. 2A), and GDP-loaded, and GTPγS-loaded HeLa protein extracts (Fig. 2B). However, significant results were not obtained using the transient transfection expression vectors in 293T cells, COS-1 cells, or HeLa cells (data not shown), likely because insufficient transfection efficiency. To circumvent this problem, stable clones of the HeLa cell line with inducible expression of HA-Dock10.1 were generated. Three out of 28 clones expressed high levels of Dock10.1 following dox withdrawal with the expected size of 250K. Clone C33 was selected for further study. Tight repression by dox was checked for C33 (Fig. 2C). First, it was checked that expression of Dock10 did not significantly affect the levels of expression of Cdc42 or Rac1. Then, it was shown that increased amounts of Cdc42 and Rac1 were precipitated by GST-PAK1-PBD from protein extracts of clone C33 following 24 h of dox withdrawal compared with control cultures in the presence of dox, indicating that expression of Dock10.1 induces activation of Cdc42 and Rac1 (Fig. 2D and Fig. 2E, respectively). Therefore, both *in vitro* interaction and activation assays suggest that Dock10 acts as a GEF for Rac1 and Cdc42.

### Effects of Dock10.1 in cell morphology and on actin cytoskeleton

**Fig. 3.**
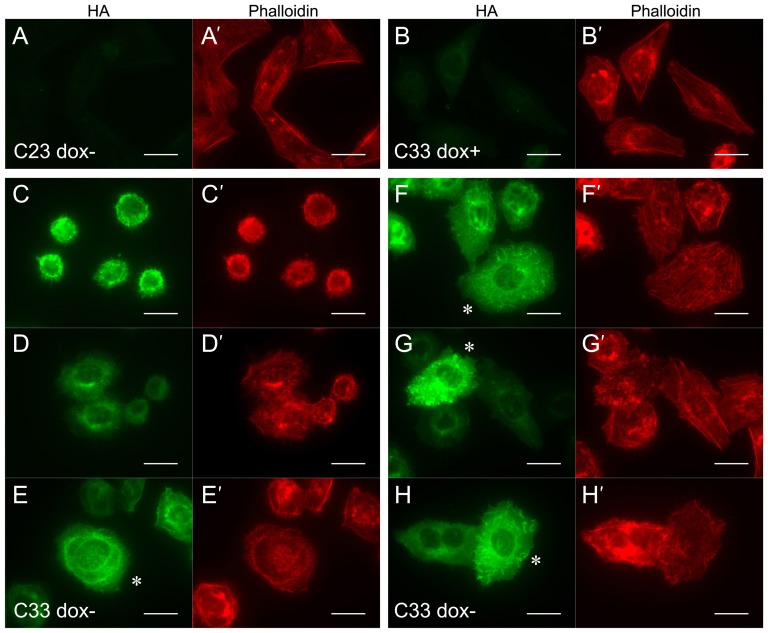
Induction of loss of cell elongation, filopodia and ruffles by Dock10.1 expression and colocalization of Dock10 with filopodia and ruffles. Immunofluorescence microscopy analysis of the negative control clone C23 cultured for 24 h free of dox, labelled with HA-FITC (A), and phalloidin-TRITC (A′), and the inducible HA-Dock10.1 HeLa clone C33 cultured for 24 h after washing the cells free of dox and reseeding with (B,B′) or without 2 ng/ml dox (C–H′) onto collagen-coated chamber slides. Polygonal elongated cells exhibit stress fibers, particularly thick at the cell edges (A′,B′). Micrographs C–D′ (all the cells displayed) and E,E′ (cell labelled with an asterisk) illustrate the highly frequent presence of abundant filopodia in HA-Dock10.1 expressor cells that have lost the normal elongated morphology of HeLa cells, and the different levels of spreading found. Micrographs F–H′ (cells labelled with an asterisk) illustrate the presence of ruffles in non-polygonal spread cells. Dock10 colocalizes with filopodia and ruffles but not with stress fibers, as exemplified in the rare non-polygonal cell with ruffles, still displaying stress fibers, shown in F,F′. Objective magnification, 60×. Dox, doxycycline. Scale bars, 25 µm.

Because small GTPases play essential roles in actin cytoskeleton dynamics, we studied the effects of Dock10 expression in cell morphology and on actin cytoskeleton. First, we examined a negative control clone, C23, and the positive clone C33 by fluorescence microscopy following double labelling with FITC-conjugated HA antibody and TRITC-conjugated phalloidin. After washing free of dox and seeding the C33 cells onto collagen-coated glass, cells attached to the surface and spread with non-polygonal, more rounded shape compared to most of the control clone C23 minus or plus dox, or the C33 cells in the presence of dox, which spread with polygonal elongated shape. In the elongated morphology, F-actin was arranged in parallel stress fibers in the direction of elongation and delineating the cell edges with polygonal shape (Fig. 3A–B′). Normal elongated HeLa cells exhibit dynamic protrusive membrane activity at the ends of the longitudinal axis of the cell, and occasional activity at the cell sides, as assessed by time lapse phase contrast analysis of cells cultured on poly-l-lysine coverslips (supplementary material Movie 1). Most non-polygonal cells from clone C33 induced to express HA-Dock10.1 were rich in filopodia (Fig. 3C–E′), and frequently developed membrane ruffles (Fig. 3F–H′; supplementary material Movie 2). Dock10.1 colocalized with F-actin in filopodia and membrane ruffles, but not in stress fibers (Fig. 3F,F′, showing a rare non-polygonal cell expressing Dock10.1 which exhibits ruffles and stress fibers). Induction of filopodia and ruffles are consistent with the proposed role of Dock10 as a GEF for Cdc42 and Rac1. These initial findings suggest that expression of Dock10.1 in HeLa cells induces loss of cell elongation.

### Effects of Dock10.1 and the Cdc42Q61L and Rac1Q61L constitutively active mutants in cell morphology

**Fig. 4.**
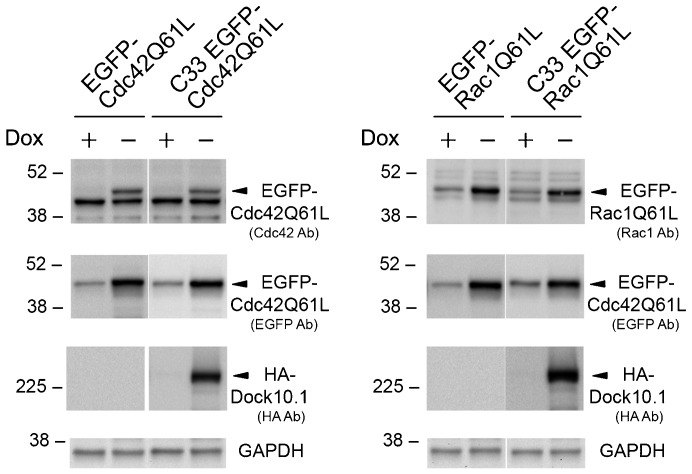
Generation of HeLa cell clones expressing EGFP-coupled, constitutively active Cdc42Q61L and Rac1Q61L mutants. Western blot analyses of the inducible EGFP-Cdc42Q61L and EGFP-Rac1Q61L mutants generated from HeLa-tTA clone and double inducible HA-Dock10.1 and EGFP-GTPaseQ61L mutants generated from HeLa clone C33. Protein extracts were performed from cells cultured in the presence and absence of 2 ng/ml dox for 24 h. The EGFP-GTPase proteins were detected with Cdc42 or Rac1 antibodies and with EGFP antibody. The position of the size markers are indicated in kDa to the left. Dox, doxycycline.

Cdc42 and Rac1 may affect actin cytoskeleton organization in different ways depending on their interactions with GEFs expressed by cells. To relate the effects produced by Dock10.1 with activation of one or another GTPase, Cdc42Q61L and Rac1Q61L mutants N-terminally fused to EGFP transfectants were generated. The small GTPase Q61L mutants are constitutively active because their inability to hydrolyze bound GTP (Rossman et al., 2005). Single inducible clones expressing the GTPases, and double clones expressing the GTPases and HA-Dock10.1, were isolated. Positive clones were detected by fluorescence, and inducible expression of the EGFP-bound GTPase mutant proteins, and of HA-Dock10.1 in clones derived from C33, was confirmed by western blot analysis (Fig. 4).

**Fig. 5.**
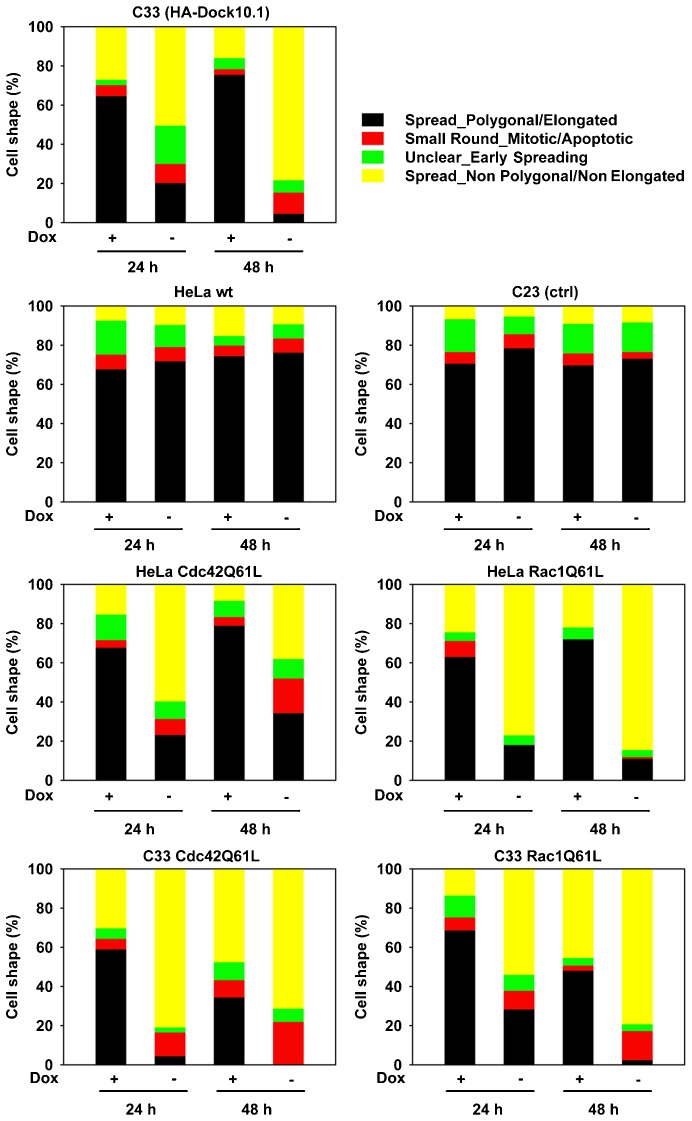
Cell morphology changes induced by expression of HA-Dock10.1 and constitutively active EGFP-Cdc42Q61L and EGFP-Rac1Q61L mutants. Proportions of polygonal elongated cells, non-polygonal/non-elongated cells, round cells, and unclear/early spreading cells counted from images of HeLa cells seeded on poly-l-lysine coating, cultured for 24 h and 48 h, and labelled with phalloidin-TRITC staining. Parental HeLa wild type (wt) cells, negative control C23, HA-Dock10.1 expressor C33, single EGFP-Cdc42Q61L and EGFP-Rac1Q61L, and double HA-Dock10.1/EGFP-Cdc42Q61L and HA-Dock10.1/EGFP-Rac1Q61L clones were compared in the presence or absence of 2 ng/ml dox. Dox, doxycycline.

The EGFP-GTPase clones were examined in parallel with the parental HeLa clone, the control clone C23, and the HA-Dock10 clone C33 cultured on poly-l-lysine-coated glass by fluorescence microscopy following labelling with phalloidin. Single clones expressing the GTPases also had less polygonal elongated cells following dox withdrawal (Fig. 5). As a rule, double clones co-expressing the GTPase mutants and the Dock10 protein, especially the double Dock10/Cdc42 clone, potentiated the presence of non-elongated cells. These results suggest that activation of both Cdc42 and Rac1 may contribute to loss of elongation of HeLa cells induced by Dock10.

### Effects of Dock10.1 and the Cdc42Q61L and Rac1Q61L mutants in cell spreading

**Fig. 6.**
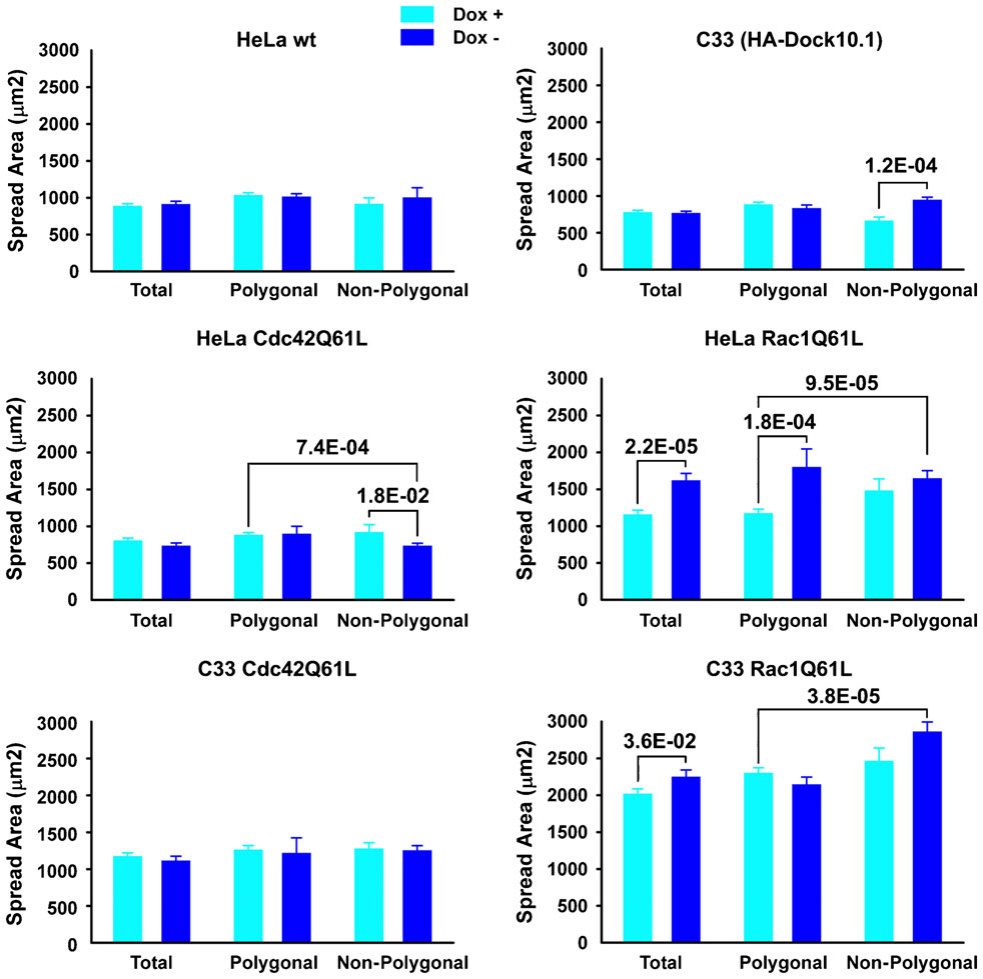
Effects of expression of HA-Dock10.1 and constitutively active EGFP-Cdc42Q61L and EGFP-Rac1Q61L mutants on cell spreading. Cell area measurements (mean±s.e.m.) of total, polygonal and non-polygonal subgroups of HeLa cells and clones seeded on poly-l-lysine coated glass, cultured for 24 h and labelled with phalloidin-TRITC staining. Parental HeLa wild type (wt) cells, HA-Dock10.1 expressor C33, single EGFP-Cdc42Q61L and EGFP-Rac1Q61L, and double HA-Dock10.1/EGFP-Cdc42Q61L and HA-Dock10.1/EGFP-Rac1Q61L clones were compared in the presence or absence of 2 ng/ml dox. P values for significant differences according to Student's t test were depicted. Dox, doxycycline.

Cell spreading was examined in clones grown on poly-l-lysine coating and labelled with phalloidin. Expression of Dock10 in C33 cells in the absence of dox induced a significant increase in the spread area of the predominant non-polygonal cells, compared to the minor fraction of non-polygonal cells in presence of dox (Fig. 6). Non-polygonal cells developed following Cdc42Q61L expression had a significant decrease in cell spreading compared to the minor fraction of non-polygonal cells, but also to the predominant fraction of polygonal cells, grown in presence of dox. The whole set of Rac1Q61L expressing cells, and both the polygonal and non-polygonal subsets, had significant increases in cell spreading in the absence of dox. HeLa cells co-expressing HA-Dock10.1 and Cdc42Q61L did not significantly alter their extension in the presence or absence of dox. However, cells co-expressing the Dock10 and Rac1 proteins had significant increases following dox withdrawal as a whole, and also for comparison between the major fraction of non-elongated cells grown in the absence of dox and the elongated cells grown in presence of dox. These results suggest that Rac1 may contribute to the increased spreading of non-elongated cells induced by Dock10.

### Effects of Dock10.1 and the Cdc42Q61L and Rac1Q61L mutants on actin cytoskeleton, filopodia and membrane ruffles

**Fig. 7.**
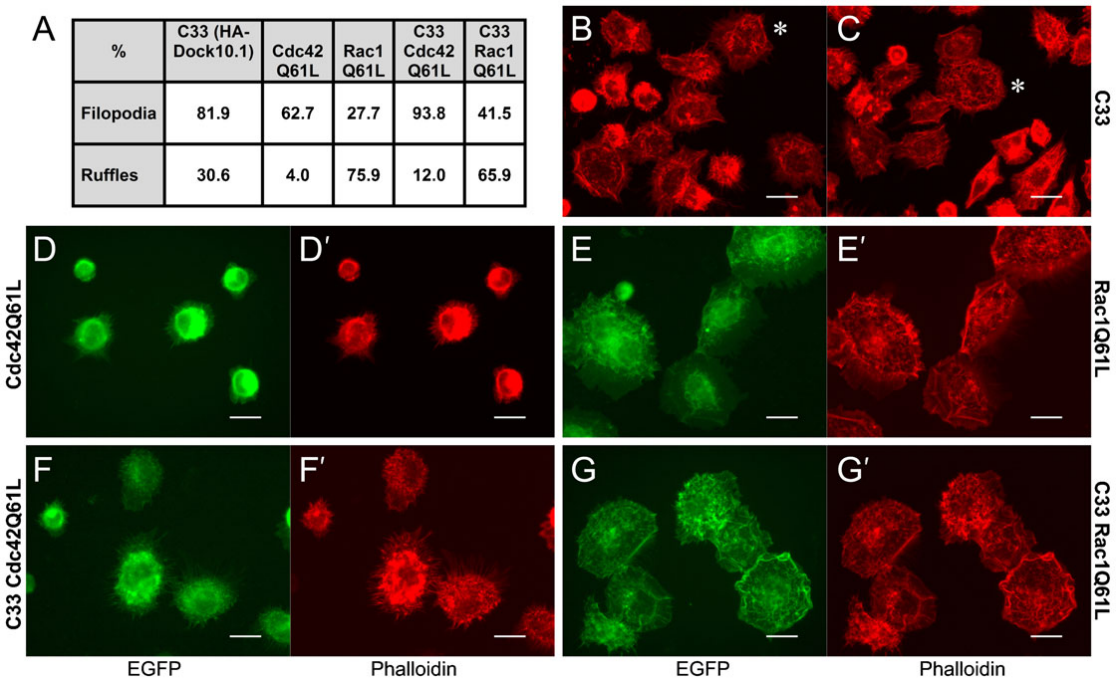
Effects of HA-Dock10.1 and constitutively active EGFP-Cdc42Q61L and EGFP-Rac1Q61L mutants on actin cytoskeleton and membrane protrusions. Immunofluorescence microscopy analysis of single HA-Dock10.1, EGFP-Cdc42Q61L, EGFP-Rac1Q61L and double HA-Dock10.1/EGFP-Cdc42Q61L and HA-Dock10.1/EGFP-Rac1Q61L clones seeded onto poly-l-lysine coating after washing the cells free of dox, cultured for 24 h, and visualized with phalloidin-TRITC (red), and EGFP (green). (A) Proportions of cells rich in filopodia or ruffles within the non-polygonal cell fraction (predominant for all these clones cultured free of dox). Micrographs B and C illustrate the extensive presence of filopodia and also the frequent presence of ruffles in non-polygonal HA-Dock10.1 C33 expressing cells (ruffles prominent in cells labelled with an asterisk). Micrographs D,D′, and F,F′, illustrate the presence of filopodia in single EGFP-Cdc42Q61L and double HA-Dock10.1/EGFP-Cdc42Q61L clones, respectively. Micrographs E,E′, and G,G′, illustrate the presence of ruffles in single EGFP-Rac1Q61L and double HA-Dock10.1/EGFP-Rac1Q61L clones, respectively. Cdc42 colocalizes with filopodia and Rac1 with ruffles. Objective magnification, 40×. Scale bars, 25 µm.

Analysis of the actin cytoskeleton in the EGFP-GTPaseQ61L clones grown poly-l-lysine coating was performed by fluorescence microscopy following phalloidin-staining (Fig. 7). HeLa wt, control clone C23, and HA-Dock10.1 expressing clone C33 had been previously studied using collagen coating (Fig. 3), and actin cytoskeleton analysis (data not shown) did not differ from that using poly-l-lysine coating. Results of the latter are summarized for the predominant non-polygonal/non-elongated fractions of C33 and EGFP-GTPaseQ61L clones in the absence of dox (Fig. 7A). C33 displayed filopodia and, frequently, ruffles. The Cdc42Q61L clone frequently exhibited filopodia. The Rac1Q61L clone extensively developed ruffles. The double Dock10/GTPase clones had mixed phenotypes where the GTPase appeared to exert a dominant role: the Dock10/Cdc42 clone had increased presence of filopodia compared to C33 and the single Cdc42Q61L clone; and the Dock10/Rac1Q61L clone had increased presence of ruffles compared with the C33 clone and increased presence of filopodia compared with the single Rac1Q61L. Representative micrographs and movies illustrate these results: extensive filopodia and frequent ruffles displayed by C33 (Fig. 7B,C; supplementary material Movie 2); frequent filopodia developed by the Cdc42Q61L clone (Fig. 7D,D′; supplementary material Movie 3); abundant ruffles exhibited by the Rac1Q61L clone (Fig. 7E,E′; supplementary material Movie 4); profuse filopodia developed by the Dock10/Cdc42Q61L (Fig. 7F,F′; supplementary material Movie 5); and last, profuse ruffles displayed by the double Dock10/Rac1Q61L clone (Fig. 7G,G′; supplementary material Movie 6). Cdc42 colocalized with filopodia and Rac1 with ruffles. These results suggest that Dock10 rearranges actin cytoskeleton by inducing filopodia through Cdc42 activation and ruffles through Rac1 activation.

## Discussion

Actin cytoskeleton dynamics is regulated by signaling through small Rho GTPases. In a gene silencing screen for GEFs, Dock10 was reported to function as a GEF for Cdc42 (Gadea et al., 2008). However, the study of the GTPase specificity of the Dock10 protein has been hindered by the lack of its full-length sequence. Our group has cloned two alternative first exon full-length isoforms of Dock10 (Alcaraz-García et al., 2011), thus enabling to perform such studies. In the present paper, we show that the Dock10 isoforms interact *in vitro* with nucleotide-free Cdc42 and Rac proteins, and that these interactions lead to increased activation of Cdc42 and Rac1 in HeLa cells. Thus, Dock10, which shares less homology with the other two Zizimin subfamily members, Dock9 and Dock11, and is 100 amino acids longer, also differs in its GTPase specificity, as these are Cdc42 specific. Dock10 is the third case among the Dock proteins, after Dock6 (Miyamoto et al., 2007) and Dock7 (Watabe-Uchida et al., 2006; Zhou et al., 2013), bearing dual specificity for Cdc42 and Rac proteins, whereas the rest of the members have unique specificity: Dock1 to Dock5 for Rac proteins (Kiyokawa et al., 1998; Nishihara et al., 2002; Kulkarni et al., 2011; Namekata et al., 2004; Hiramoto et al., 2006; Vives et al., 2011) and Dock8, Dock9, and Dock11, for Cdc42 (Harada et al., 2012; Meller et al., 2002; Nishikimi et al., 2005; Lin et al., 2006).

We have studied function of the Dock10.1 isoform by inducing its overexpression in adherent HeLa cells growing in planar surfaces, including tissue culture plastic, and collagen and poly-l-lysine coated glass. Our main findings are that the Dock10 protein induces a morphological change from polygonal elongated to more rounded, non-polygonal cells, which develop abundant filopodia, frequent membrane ruffles, and moderately increase their spread area in contact with the substrate. Loss of elongation was consistent with previous results by Gadea and coworkers, showing that Dock10 silencing induces the opposite effect, i.e., a change from rounded to elongated, in a melanoma cell line in three-dimensional environment (Gadea et al., 2008). In our paper, HeLa cells expressing the Cdc42 and Rac1 constitutively active mutants also induce loss of cell elongation, and this phenotype is sustained or even potentiated by co-expression of Dock10 and the GTPases, especially Cdc42. These observations are consistent with previously reported observations in Cdc42 null fibroblasts, which present with spindle shape (Czuchra et al., 2005), and in Rac1 null fibroblasts, which present with elongated shape and are defective in lamellipodia and ruffle formation (Vidali et al., 2006). Therefore, Dock10.1 may induce loss of cell elongation through both Cdc42 and Rac1.

The morphological change induced by Dock10.1 is not accompanied by a global change in the cell area in contact with the surface, suggesting that transition from elongated to non-elongated does not imply a loss of spreading capacity. In fact, our results even show that non-elongated cells expressing Dock10 spread more than non-elongated cells under Dock10 repression by dox. Previous reports indicate that Cdc42 and Rac1 play roles in cell spreading (Price et al., 1998; Wells et al., 2004; Czuchra et al., 2005). Our results show that constitutively active Cdc42 does not induce a significant global change of cell spreading, though moderately reduce extension of non-elongated cells, and no changes are observed in the double Dock10/Cdc42 cells. In contrast, constitutively active Rac1 potentiates surface expansion, and this observation is reproduced by the double Dock10/Rac1 cells. We note that the clones may have leaky expression in the presence of dox, which may generate baseline differences. Thus, the double Dock10/Rac1 cells display the largest spread area between our clones in presence of dox, possibly due to leaky expression of Rac1 and Dock10. These results suggest that the increased spreading capacity of non-elongated cells expressing Dock10 may be mediated by Rac1.

Protrusions induced by Dock10.1, filopodia and membrane ruffles, are consistent with activation of Cdc42 and Rac1, respectively. Indeed, the constitutively active Cdc42 mutant induces filopodia, and the constitutively active Rac1 mutant, ruffles. Our data suggest that the constitutively active GTPase proteins act in a dominant fashion in the double clones, as the double Dock10/Cdc42 cells extensively exhibit filopodia while showing reduced ruffling activity, and the double Dock10/Rac1 cells extensively display ruffles while showing a substantial decrease of filopodia.

In summary, using an inducible gene expression system in a cancer adherent cell line, we present here the first cellular model for studying Dock10 function by means of its overexpression. Our data make firm the previously reported roles of Dock10 in loss of cell elongation and Cdc42 activation, and support new roles for Dock10, in Rac1 activation, induction of filopodia and membrane ruffles. Our stable Dock10.1 inducible transfectant cell lines will be valuable for investigating the functions of this regulator of small GTPases in different environments.

## Supplementary Material

Supplementary Material
