# Supplementary material for: Dock10, a Cdc42 and Rac1 GEF, induces loss of elongation, filopodia, and ruffles in cervical cancer epithelial HeLa cells
Source: Biol Open. 2015 Apr 10;4(5):627–35. doi: 10.1242/bio.20149050 (PMC4434814; doi:10.1242/bio.20149050)
Supplement: Supplementary Material [file supp_4_5_627__index.html]

Dock10, a Cdc42 and Rac1 GEF, induces loss of elongation, filopodia, and ruffles in cervical cancer epithelial HeLa cells — Dock10, a Cdc42 and Rac1 GEF, induces loss of elongation, filopodia, and ruffles in cervical cancer epithelial HeLa cells — Supplementary Material 

# Dock10, a Cdc42 and Rac1 GEF, induces loss of elongation, filopodia, and ruffles in cervical cancer epithelial HeLa cells

## bio.20149050 Supplementary Material

**Files in this Data Supplement:**

- Supplementary Material - Natalia Ruiz-Lafuente et al. doi: 10.1242/bio.20149050
- Movie 1 - **HeLa cells (wt) seeded on a poly-L-lysine-coated coverslip placed into a 24-well plate were cultured for 24 h, and then mounted in a heated stage chamber.** Phase contrast images were registered every 20 s for 10 min in a Nikon Eclipse T*i* inverted microscope. Protrusive membrane activity at the cell vertices is depicted by arrows.
- Movie 2 - **HeLa cell clone C33 cells expressing HA-Dock10.1 processed as for supplementary material Movie 1.** Movie shows a non-elongated flattened cell (center) displaying filopodia and ruffles. Ruffles are indicated by arrows.
- Movie 3 - **HeLa cell clone expressing EGFP-Cdc42Q61L processed as for previous movies.** Phase contrast (left) and EGFP (right) images were registered. Non-elongated cells display filopodia.
- Movie 4 - **HeLa cell clone expressing EGFP-Rac1Q61L, processed as for previous movies.** Phase contrast (top) and EGFP (bottom) images were registered. Non-elongated flattened cells exhibit extensive ruffling activity.
- Movie 5 - **HeLa cell clone HeLa cell clone co-expressing HA-Dock10.1 and EGFP-Cdc42Q61L, processed as for previous movies.** Phase contrast (left) and EGFP (right) images were registered. Non-elongated cells profusely display filopodia.
- Movie 6 - **HeLa cell clone HeLa cell clone co-expressing HA-Dock10.1 and EGFP-Rac1Q61L, processed as for previous movies.** Phase contrast (left) and EGFP (right) images were registered. Movie shows a non-elongated flattened cell exhibiting extensive ruffling activity.
- Table S1 - Plasmids used in this work.
