# Supplementary material for: Dock10, a Cdc42 and Rac1 GEF, induces loss of elongation, filopodia, and ruffles in cervical cancer epithelial HeLa cells
Source: Biol Open. 2015 Apr 10;4(5):627–35. doi: 10.1242/bio.20149050 (PMC4434814; doi:10.1242/bio.20149050)
Supplement: Supplementary Material [file supp_bio.20149050_bio.20149050-s1.pdf]

Biology Open (2015) 000, 1–9 doi:10.1242/bio.20149050

Supplementary Material
Natalia Ruiz-Lafuente et al. doi: 10.1242/bio.20149050

Table S1. See supplementary webpage

Table S2. Antibodies used in this work

Protein target (clone)	Dilution (units)	Dilution (ratio)	Source	Used for	Reference	Manufacturer
Dock9	0.04 µg/ml	1:5000	Rabbit	WB	A300-530A	Bethyl Laboratories
Dock10	0.2 µg/ml	1:5000	Rabbit	WB	A301-305A	Bethyl Laboratories
Dock10.2		1:5000	Rabbit	WB	Yelo, et al., 2008	Our lab
Dock11	0.2 µg/ml	1:5000	Rabbit	WB	A301-639A	Bethyl Laboratories
Cdc42	0.25 µg/ml	1:1000	Mouse	WB	ACD03	Cytoskeleton
Rac1	0.5 µg/ml	1:1000	Mouse	WB	ARC03	Cytoskeleton
Rac (23A8)	1 µg/ml	1:1000	Mouse	WB	05-389	Upstate Technologies
Flag tag (M2)	0.2 µg/ml	1:5000	Mouse	WB	F3165	Sigma-Aldrich
HA tag (3F10)	1 µg/ml	1:100	Rat	IF	11 867 423 001	Roche Applied Science
HA-tag-HRP (3F10)	5 ng/ml	1:5000	Rat	WB	12 013 819 001	Roche Applied Science
GAPDH-HRP (FL-335)	0.2 µg/ml	1:1000	Rabbit	WB	sc-25778	Santa Cruz Laboratories
EGFP (JL-8)	1 µg/ml	1:1000	Mouse	WB	632381	Clontech
Anti-mouse-HRP	0.5 µg/ml	1:2000	Goat	WB	P0447	Dako
Anti-rabbit-HRP	0.17 µg/ml	1:2000	Swine	WB	P0399	Dako
Anti-rat-Alexa Fluor 488	2 µg/ml	1:100	Goat	IF	A11006	Invitrogen

Biology Open (2015) 000, 1–9 doi:10.1242/bio.20149050

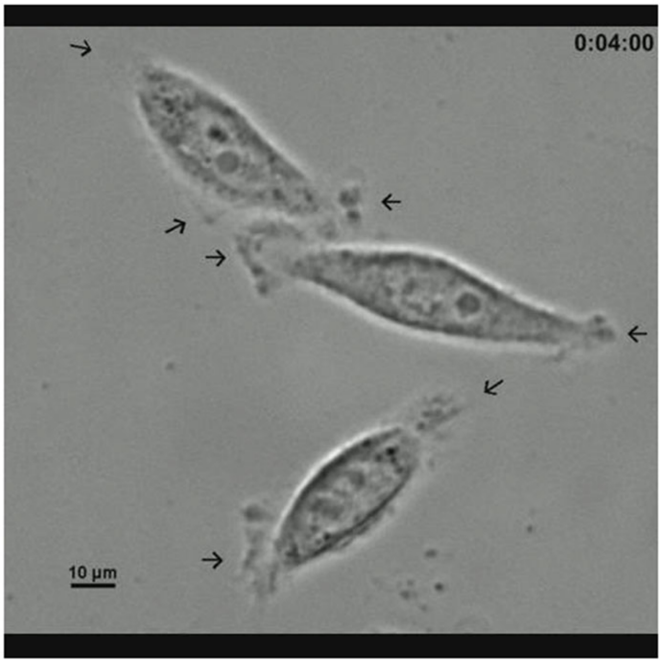

Movie 1. HeLa cells (wt) seeded on a poly-L-lysine-coated coverslip placed into a 24-well plate were cultured for 24 h, and then mounted in a heated stage chamber. Phase contrast images were registered every 20 s for 10 min in a Nikon Eclipse T*i* inverted microscope. Protrusive membrane activity at the cell vertices is depicted by arrows.

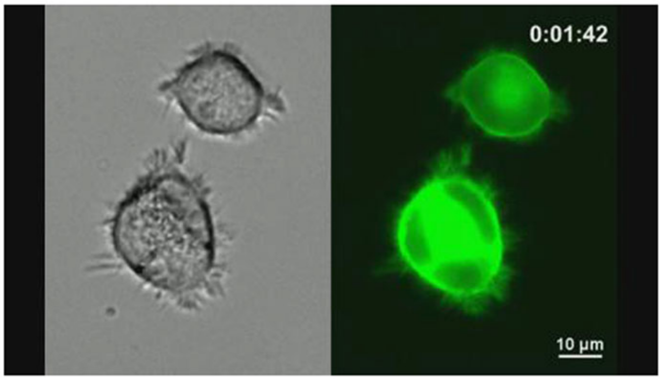

Movie 3. HeLa cell clone expressing EGFP-Cdc42Q61L processed as for previous movies. Phase contrast (left) and EGFP (right) images were registered. Non-elongated cells display filopodia.

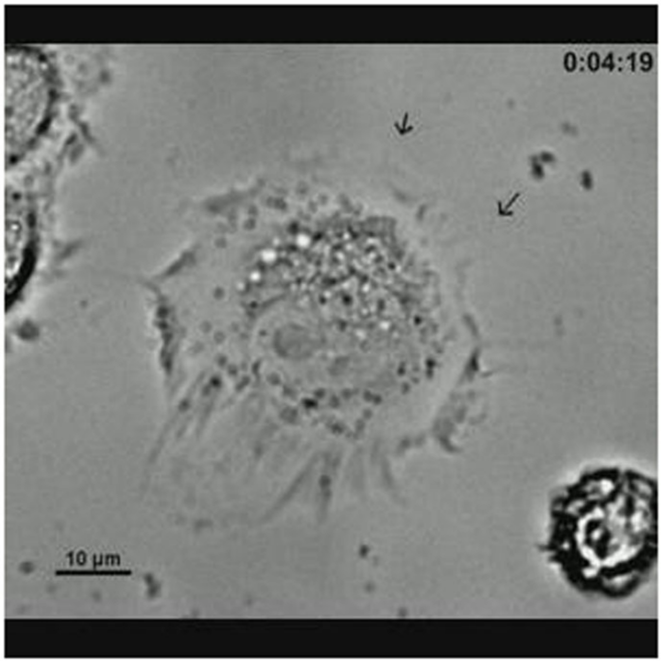

Movie 2. HeLa cell clone C33 cells expressing HA-Dock10.1 processed as for supplementary material Movie 1. Movie shows a non-elongated flattened cell (center) displaying filopodia and ruffles. Ruffles are indicated by arrows.

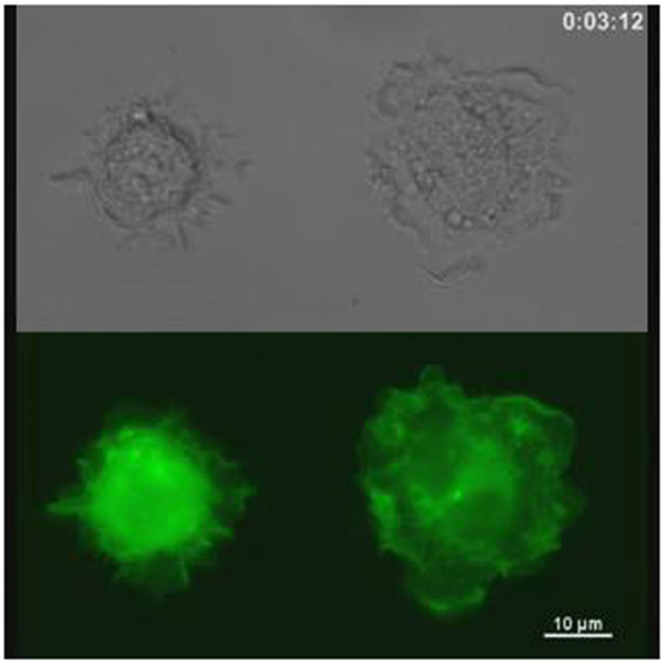

Movie 4. HeLa cell clone expressing EGFP-Rac1Q61L, processed as for previous movies. Phase contrast (top) and EGFP (bottom) images were registered. Non-elongated flattened cells exhibit extensive ruffling activity.

Biology Open

Biology Open (2015) 000, 1–9 doi:10.1242/bio.20149050

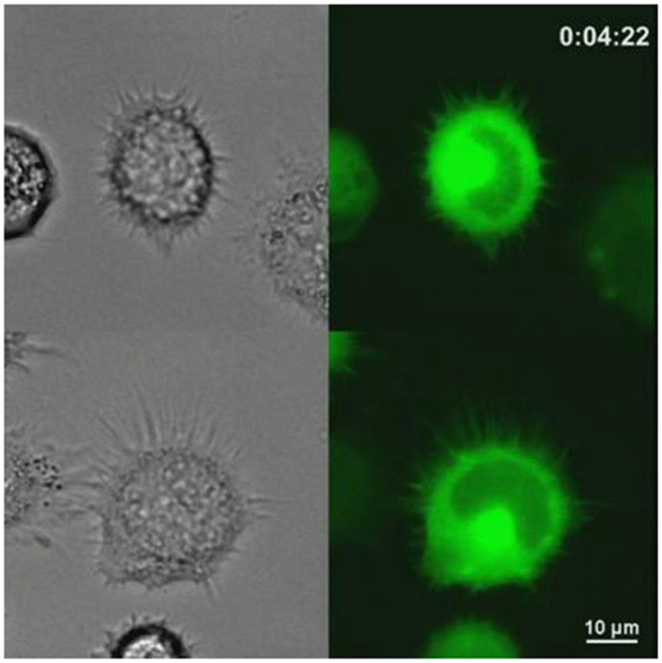

Movie 5. HeLa cell clone HeLa cell clone co-expressing HA-Dock10.1 and EGFP-Cdc42Q61L, processed as for previous movies. Phase contrast (left) and EGFP (right) images were registered. Non-elongated cells profusely display filopodia.

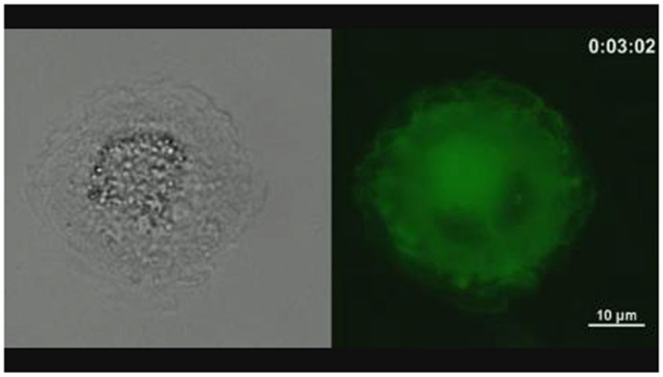

Movie 6. HeLa cell clone HeLa cell clone co-expressing HA-Dock10.1 and EGFP-Rac1Q61L, processed as for previous movies. Phase contrast (left) and EGFP (right) images were registered. Movie shows a non-elongated flattened cell exhibiting extensive ruffling activity.
