# Supplementary material for: Dock10, a Cdc42 and Rac1 GEF, induces loss of elongation, filopodia, and ruffles in cervical cancer epithelial HeLa cells
Source: Biol Open. 2015 Apr 10;4(5):627–35. doi: 10.1242/bio.20149050 (PMC4434814; doi:10.1242/bio.20149050)
Supplement: Supplementary Material [file supp_bio.20149050_Table_S1.docx]

**Table S1. Plasmids used in this work**

| **Vector** | **NCBI acc. no.** | **Generation** | |
| --- | --- | --- | --- |
| **Bacterial production of N-terminal GST-fusion recombinant protein** | | | |
| pGEX-Cdc42 | NM_044472.2 | | RT-PCR amplification of Cdc42 from PBMC using 5’-TTCCCGGGGCAGACAATTAAGTGTGTTG-3’ and 5’- TTGCGGCCGCTTAGAATATACAGCACTTCC-3’, and ligation into SmaI/NotI sites of pGEX-4T-1 (GE Healthcare) |
| pGEX-Rac1 | NM_006908.4 | | RT-PCR amplification of Rac1 from PBMC using 5’- TTCCCGGGGCAGGCCATCAAGTGTGTGG-3’ and 5’- TTGCGGCCGCTTACAACAGCAGGCATTTTC-3’ and ligation into SmaI/NotI sites of pGEX-4T-1 |
| pGEX-Rac2 | NM_002872.4 | | PCR amplification of YFP-Rac2 (Addgene plasmid 11393, Hoppe&Swanson, 2004) using 5’- TTGAATTCATGCAGGCCATCAAGTGTGTGGTGG -3’ and 5’-TTGCGGCCGCCTAGAGGAGGCTGCAGGCGCGCTTC-3’, and ligation into EcoRI/NotI sites of pGEX-4T-1 |
| pGEX-Rac3 | NM_005052.2 | | PCR amplification of LZRS-MS-IRES-ZEO/pBR-Rac3 (Hajdo-Milasinović et al., 2007) using 5’- TTGAATTCATGCAGGCCATCAAGTGCGTGGTGG-3’ and 5’- TTGCGGCCGCCTAGAAGACGGTGCACTTCTTCCCC-3’, and ligation into EcoRI/NotI sites of pGEX-4T-1 |
| pGEX-RhoA | NM_001664.2 | | RT-PCR amplification of RhoA from PBMC using 5’- GAATTCATGGCTGCCATCCGGAAGAAAC-3’ and 5’- TTGCGGCCGCTTAGAATATACAGCACTTCC-3’, and ligation into EcoRI/NotI sites of pGEX-4T-1 |
| pGEX-RhoD | NM_014578.3 | | PCR amplification of EGFP-hRhoD (Addgene plasmid 23235, Roberts et al, 2008) using 5’-TTGAATTCATGACGGCGGCCCAGGCCGCGGGTG-3’ and 5’-TTGCGGCCGCTCAGGTCACCACGCAAAAGCCCTGG-3’, and ligation into EcoRI/NotI sites of pGEX-4T-1 |
| pGEX-RhoF-SAAX | NM_019034.2 | | PCR amplification of pGEX-2T-RhoF (gift of Harry Mellor, University of Bristol, UK) introducing amino acid change C208S using 5’-TTGAATTCATGGATGCCCCCGGGGCCCTGGCCC-3’ and 5’-TTGCGGCCGCTCAGAGCAGCAGGGAGAGCCGGCGC-3’, and ligation into EcoRI/NotI sites of pGEX-4T-1 |
| pGEX-RhoG-SAAX | NM_001665.3 | | RT-PCR amplification of RhoG from PBMC introducing amino acid change C188S using 5’-TTGAATTCATGCAGAGCATCAAGTGCGTGGTGG-3’ and 5’-TTGCGGCCGCCTACAAGAGGATGGAGGACCGCCCA-3’, and ligation into EcoRI/NotI sites of pGEX-4T-1 |
| pGEX-RhoJ | NM_020663.3 | | RT-PCR amplification of RhoJ from PBMC using 5’-TTGAATTCATGAACTGCAAAGAGGGAACTGACA-3’ and 5’- TTGCGGCCGCTCAGATAATTGAACAGCAGCTGTGA-3’, and ligation into EcoRI/NotI sites of pGEX-4T-1 |
| pGEX-2T-RhoQ | NM_012249.3 | | Neudauer et al., 1998 |
| pGEX-PAK1 | NM_002576.3 | | RT-PCR amplification of PAK1-PBD (amino acids 67-150) from human brain using 5’-TTCCCGGGGAAGAAAGAGAAAGAGCGGCC-3’ and 5’- TTGCGGCCGCTCAAGCTGACTTATCTGTAAAGC-3’, and ligation into SmaI/NotI sites of pGEX-4T-1 |
| **Eukaryotic expression, transient** | | | |
| pSG5a |  | | New MCS (AgeI-EcoRI-NotI) for pSG5 vector (Stratagene) by PCR amplification of pSG5 with 5’-TTGAATTCGCGGCCGCTATTAAAGCAGAACTTGTTTATTGCA-3’ and 5’- TTGAATTCACCGGTTATAGTGAGTCGTATTACAATTCT-3’, EcoRI digestion, and religation of vector |
| pSG5b |  | | New MCS (BamHI-EcoRI-SacII) for pSG5 vector by PCR amplification of pSG5 with 5’-TTGAATTCGGATCCTATTAAAGCAGAACTTGTTTATTGCA 3’ and 5’-  TTGAATTCCCGCGGTATAGTGAGTCGTATTACAATTCT-3’, EcoRI digestion, and religation of vector |
| pEF-FLAG-DOCK9 | NM_015296.2 | | Meller et al., 2004 |
| pSG5- DOCK10.1 | NM_014689 | | Subcloning of DOCK10.1 from pJAG4-DOCK10.1 (this work) into AgeI/NotI sites of pSG5a |
| pSG5-HA-DOCK10.1 | NM_014689 | | Subcloning of HA-DOCK10.1 from pJAG4-HA-DOCK10.1 (this work) into the AgeI/NotI sites of pSG5a |
| pSG5- DOCK10.2 | NM_001290263.1 | | Subcloning of DOCK10.2 from pJAG4-DOCK10.2 (this work) into the AgeI/NotI sites of pSG5a |
| pSG5-HA-DOCK10.2 | NM_001290263.1 | | PCR amplification of pJAG4-HA-DOCK10.2 (this work) using 5’-TTACCGGTAGCGCCGCCATGGAG-3’ and 5’-TGTGAAGGAAGCTTCTCTGGT-3’, excision of AgeI/EcoRV fragment of pSG5-DOCK10.2 (this work), and ligation of PCR fragment into AgeI/EcoRV sites |
| pSG5-DOCK11 | NM_144658.3 | | Subcloning of DOCK11 from pJEF4-DOCK11 (this work) into SacII/BamHI sites of pSG5b |
| pSG5-HA-DOCK11 | NM_144658.3 | | Subcloning of HA-DOCK11 from pJEF4-HA-DOCK11 (this work) into SacII/BamHI sites of pSG5b |
| **Eukaryotic expression, stable inducible** | | | |
| pUHD-15-1-Puro |  | | Gift from Berthold Henglein (Institut Curie, Paris, France) (Bernardo et al, 2007) |
| pUHC-13-3 |  | | Gossen&Bujard, 1992 |
| pJAG1 |  | | New MCS (SacII-EcoRI-AflII-Eco47III-SnaBI-SpeI-SalI-MluI-XbaI-EcoRV-AgeI-NheI-NotI-ApaI-SbfI-BamHI) for pJEF4 , gift of J.E. Floettmann & M. Rowe (University of Wales, Cardiff, UK) (Parrado et al, 2000) by insertion of synthetic oligonucleotide, ligated following excision of EcoRI/BamHI fragment |
| pJAG2 |  | | Exchange of Neomycin for Zeocin resistance in pJAG1 (this work) by excision of Neomycin resistance cassette with XhoI, PCR amplification of Zeocin resistance cassette of pSV40-Zeo2 (Invitrogen) using 5’-AGCTCGAGGGTGTGGAAAGT-3’ and 5’-TTCTCGAGAGACATGATAAGATACATTG-3’, and ligation into XhoI site |
| pJAG4 |  | | Modification of MCS of pJAG1 (this work) by excision of Eco47III//EcoRV fragment and religation of vector |
| pJAG4- DOCK10.1 | NM_014689 | | PCR amplification of pCR2.1-hDOCK10.1 (Alcaraz-García et al., 2011) using 5’-TTACCGGTTGACCGGCGATGGCCGGTGA-3’ and 5’-TAGCGGCCGCCCTCAGACTTCAGCACTA-3’, and ligation into AgeI/NotI sites of pJAG4 |
| pJAG4-HA-DOCK10.1 | NM_014689 | | Insertion of HA tag into pJAG4-DOCK10.1 (this work) by excision of AgeI/EcoRV fragment from pJAG4-DOCK10.1, PCR amplification of pJAG4-DOCK10.1 using 5’-TTACCGGTAGCGCCGCCATGGAGTACCCATACGACGTACCAGATTACGCTGCCGGTGAGCGG-3’ and 5’-TGTGAAGGAAGCTTCTCTGGT-3’, and ligation of PCR fragment into AgeI/EcoRV sites |
| pJAG4-DOCK10.2 | NM_001290263.1 | | PCR amplification of pCR2.1-hDOCK10.2 (Alcaraz-García et al., 2011) using 5’-TTACCGGTAGCAATACGATGAGTTTTC-3’ and 5’-TGTGAAGGAAGCTTCTCTGGT-3’, excision of AgeI/EcoRV fragment from pJAG4-DOCK10.1, and ligation of PCR fragment into AgeI/EcoRV sites |
| pJEF4-DOCK10.2* | Genbank EU236710.1 | | Old version of pJEF4-DOCK10.2 (*; contains mutations) by RT-PCR amplification of human B cells using 5’-TTCCGCGGAGCAATACGATGAGTTTTC-3’ and 5’-AACCGCGGTCAGACTTCAGCACTAGATG-3’ and ligation into SacII site of pJEF4 |
| pJEF4-HA-DOCK10.2* | Genbank EU236710.1 | | Insertion of HA tag in old version of pJEF4-DOCK10.2 (*; contains mutations) by PCR amplification of pJEF4-DOCK10.2* using 5’-ATCGCCTGGAGACGCCATCCACG-3’ and 5’-GTTCGTCTCTTCTTCCAAAATTCACTGGGCTCCCGTTTAAAAACCTTCCCTCGAAAACTAGCGTAATCTGGTACGTCGTATGGGTACTCCATGGCGGCGCTCCGCGGAGGCTGGATCGGTC-3’, excision of EspI/EspI fragment from pJEF4-DOCK10.2*, and ligation of PCR fragment into EspI site |
| pJAG4-HA-DOCK10.2 | NM_001290263.1 | | Insertion of HA tag into pJAG4-DOCK10.2 (this work) by subcloning EspI/EspI fragment of pJEF4-HA-DOCK10.2* into pJAG4-DOCK10.2 |
| pJEF4-DOCK11 | NM_144658.3 | | Deletion of HA tag from pJEF4-HA-DOCK11 (this work) by excision of SacII/ApaI fragment, PCR amplification of pJEF4-HA-DOCK11 using TTCCGCGGGCCGCTGCCATGGCCGAAGT and CACACCACCCTTCTGAGAAC, and ligation of PCR fragment into SacII/ApaI sites |
| pJEF4-HA-DOCK11 | NM_144658.3 | | PCR amplification of pKH3-DOCK11 (Lin et al, 2006) using 5’-TTCCGCGGAGCGCCGCCATGGAGTACCCATACGACGTACCAGATTACGCTGCCGAAGTGCGCAAATTCAC-3’ (containing HA tag) and 5’-TTGGATCCTCACACTTCAGCGTATCTTG-3’, and ligation into SacII/BamHI sites of pJEF4; aminoacid substitution R727H by excision of AccIII/Eco47III internal fragment, RT-PCR amplification of PBMC using GGAGACGGTAGAAACAGCAC and TGTGCTGGTATCTTGTGTCA, and ligation of PCR fragment into AccIII/Eco47III sites. |
| pJAG2-EGFP- Cdc42Q61L | NM_001791.3 | | PCR amplification of pcDNA3-EGFP-Cdc42-Q61L (Addgene plasmid 12986, Subauste et al., 2000) using 5’-TTCCGCGGGCCGCCACCATGGTGAGCAAGG-3’ and 5’-TTGGATCCTCATAGCAGCACACACCTGC-3’, and ligation into SacII/BamHI sites of pJAG2 |
| pJAG2-EGFP-Rac1Q61L | NM_006908.4 | | PCR amplification of pcDNA3-EGFP-Rac1-Q61L (Addgene plasmid 12891, Subauste et al., 2000) using 5’-TTCCGCGGGCCGCCACCATGGTGAGCAAGG-3’ and 5’-TTGGATCCTTACAACAGCAGGCATTTTC-3’, and ligation into SacII/BamHI sites of pJAG2 |
